# Muscle specific differences in the regulation of myogenic differentiation in chickens genetically selected for divergent growth rates

**DOI:** 10.1016/j.diff.2011.05.012

**Published:** 2011-10

**Authors:** Sara L. Al-Musawi, Francesca Lock, Biggy H. Simbi, Stéphanie A.M. Bayol, Neil C. Stickland

**Affiliations:** Department of Veterinary Basic Sciences, the Royal Veterinary College, Royal College Street, London, NW1 0TU, United Kingdom

**Keywords:** MyoD, Myogenin, IGF-I, Muscle, Chick embryo, Movement

## Abstract

With the human population predicted to reach 9 billion by 2050, increasing food supplies while maintaining adequate standards of animal welfare has become a global priority. In the poultry industry, broilers are genetically selected for greater pectoral but not leg muscularity yield leading to leg disorders and thereby welfare issues. It is known that the pectoralis major of broilers contains more muscle fibres of larger diameters than egg-layers but little is known about the leg gastrocnemius muscle cellular characteristics. As muscle fibre numbers are set by hatch, the molecular regulation of myogenesis was investigated in pectoral (selected) and gastrocnemius (unselected) muscles of chick embryos to help explain diverging post-hatch phenotypes. Results showed that broilers were more active from embryonic day (ED) 8 and heavier from ED12 to 18 than layers. The pectoral muscle of broilers exhibited increased myoblast proliferation on ED15 (raised myonuclei, MyoD and PCNA) followed by increased differentiation from ED16 (raised myogenin, IGF-I) leading to increased muscle fibre hyperplasia and mass by ED18 compared to layers. In the gastrocnemius muscle of broilers, cell proliferation was also raised up to ED15 accompanied by increased PCNA, MyoD and IGF-I mRNAs. However, from ED16, myogenin and IGF-I mRNAs were similar to that of layers and PCNA was reduced leading to similar fibre area, nuclei numbers and muscle mass at ED18. We conclude that genetic selection for enhanced post-hatch pectoral muscle growth has altered the temporal expression of IGF-I and thereby myogenin transcription affecting cellular characteristics and mass by hatch in a muscle specific manner. These observations should help develop intervention strategies aimed at improving leg muscle strength and thereby animal welfare to meet growing consumer demand.

## Highlights

► We compared myogenesis in chicken strains of divergent post-hatch growth rates. ► Broilers showed raised embryonic activity from ED8 and were heavier by hatch. ► mRNA levels of MyoD, myogenin and IGF-I were altered in a muscle-dependant manner. ► Cellular characteristics were altered accordingly.

## 1 Introduction

The human population is predicted to reach 9 billion by 2050, therefore increasing food supplies while maintaining diversity, quality and adequate standards of animal welfare has become a global priority. Since around the 1950s, the poultry industry has actively genetically selected meat-type chickens for faster post-hatch growth rates and greater yield of breast but not leg meat to increase financial profitability and meet consumer demand (Arthur and Albers, 2003, Guernec et al., 2003). Since then, the growth rate of modern broilers has almost quadrupled and the relative size of the pectoral muscle has dramatically increased at the expense of leg strength, leading to leg disorders, impaired ability to walk and thereby major welfare issues (Arthur and Albers, 2003). It is known that the pectoralis major of broilers contains more muscle fibres of larger diameters than layers (Scheuermann et al., 2004), but little is known about how intense selective breeding has affected leg muscle cellular characteristics. Understanding the developmental differences between selected breast and unselected limb muscles is a necessary step for the development of intervention strategies aimed at improving leg muscle strength and thereby animal welfare in the current climate of rapidly increasing demand of food supplies and food security concerns.

Given that unlike the meat-broiler breed, egg-layer chickens do not suffer from leg deformities and lameness, we used these two strains to examine possible developmental differences between the selected pectoral and unselected gastrocnemius leg muscles of broilers, which may explain the diverging post-natal growth rates and muscle phenotypes at the forefront of welfare issues. Almost all vertebrate skeletal muscles develop in a multistep process whereby pluripotent stem cells derived from the paraxial mesoderm give rise to myogenic precursors, which commit to the formation and maturation of myoblasts. In response to highly orchestrated cues, proliferating myoblasts permanently withdraw from the cell cycle, terminally differentiate and eventually fuse into multinucleated myotubes (for review see Christ and Ordahl, 1995). During the 3-week period of chick embryonic development, myogenesis occurs in two waves, sequentially generating two populations of myotubes. The primary myotubes, with centrally located nuclei, form between embryonic days (EDs) 4 to 7 and the secondary myotubes, which use the primaries as scaffolding, form between ED8 and 16 (Crow and Stockdale, 1986, Lee et al., 2004). By hatch (ED21), the total fibre numbers are typically set (Smith, 1963). Subsets of undifferentiated muscle precursor cells, namely the satellite cells, remain associated with the developing muscle fibres and play prominent roles in adult muscle regeneration and growth (Moss, 1968).

The activation of the myogenic programme and specification of muscle specific gene expression in the developing embryo is controlled by a family of 4 basic helix–loop–helix (bHLH) transcription factors, collectively known as the myogenic regulatory factors (MRFs). Originally discovered for their ability to confer a myogenic fate on non-muscle cells (Davis et al., 1987), these factors can also activate their own and each other's expression (for review, see Weintraub, 1993). Of these factors, MyoD and Myf-5 play early roles in the determination of muscle precursor cells to the myogenic lineage. Although both are expressed in proliferating myoblasts, evidence also suggests Myf-5 functions more towards proliferation whilst MyoD prepares myoblasts for efficient differentiation (Ishibashi et al., 2005). Adult satellite cells are additionally shown to originate from MyoD^+^ cell (Kanisicak et al., 2009) which highlights the relationship between commitment of myogenic progenitors and future satellite cell function. In contrast, the secondary MRF myogenin is required for the committed cells to terminally differentiate into myocytes and mature into fibres (Andres and Walsh, 1996).

MRFs are extremely sensitive to growth factors. Of these, the insulin-like growth factor (IGF) system has been implicated in the control of skeletal muscle development and growth, during embryogenesis, post-natal muscle hypertrophy and regeneration (Florini et al., 1996, Duclos, 2005). The IGF system is a complex network of ubiquitous ligands (IGF-I and IGF-II), tyrosine kinase receptors, binding proteins and proteases that regulate crucial biological outcomes. The IGFs are unique in that they exert pleiotrophic effects, capable of independently stimulating myoblast proliferation, whilst inhibiting differentiation and then switching to promoting cell cycle withdrawal and differentiation (Florini et al., 1986). IGF-I has also been shown to mediate the mechano-signal transduction of muscle growth in response to increased activity levels (Tidball, 2005).

Previous work in our laboratory has shown that increasing embryonic movement either through neuromuscular stimulation or by manipulating egg incubation temperature resulted in muscle fibre hyperplasia (Hammond et al., 2007, McEntee et al., 2006). We hypothesise that broilers will also exhibit increased embryonic activity related to differing expression levels of MRFs and IGFs. The mRNA expression patterns of key MRFs (MyoD and myogenin) and IGF-I were therefore examined in broilers and layers together with measurements of muscle mass and cellularity (nuclei number, fibre number and fibre size).

## 2 Methods

### 2.1 Animals

A total of 112 fertilised chicken eggs were used for this study. Of these eggs, 56 were of either broiler (Ross; Faccenda Hatchery, Essex, UK) or layer (White Leghorn; Joice and Hill Poultry Ltd, Norfolk, UK) strain. All eggs were incubated in the same manner: eggs were positioned horizontally and equally distributed on fibre egg trays in an LMS 301 forced draft incubator (Wolf Laboratories, York, UK). Incubations were carried out in the dark at 37.5 °C with relative humidity of ∼60–70%. A high precision Squirrel Logger (Grant Instruments Ltd., Cambridge, UK) monitored the temperature within the incubator, with 10 probes each recording the temperature every 5 min. Daily egg rotations ensured normal embryonic development and prevented malpositioning within the egg (Tona et al., 2003).

Analyses were carried out in 4 eggs per ED and per strain between ED5 and ED18 (112 eggs in total). Quantification of total body movements was carried out between ED5 and 11 inclusively in 4 eggs per strain and per ED (56 eggs in total). The analysis did not extend beyond ED11 as the egg shell size was considered to introduce physical constraint on the rapidly-growing chick (Sharp et al., 1999). Motility measurements were carried out as follows: an egg was removed from the incubator, placed in an insulated support and ‘windowed’. A stereomicroscope LED spot light was placed at the blunt end of the egg to illuminate the inside and total movement recordings made over a 5 min period were counted (Hammond et al., 2007). Amnion contractions, which promoted a swinging motion of the embryo, were excluded (Oppenheim, 1966). Motility measurements were carried out by one researcher, with randomly selected samples tested by an independent researcher. Following each motility measurement, chicks from ED5 to 11 were freed from their eggs and weighed. Chicks older than ED7 were killed by decapitation.

### 2.2 Sampling

Chicks from ED13 to 18 were weighed; their pectoral and gastrocnemius muscles as well as liver and heart were removed and weighed. The left leg gastrocnemius and left portion of the pectoral muscle (muscle cut down the sternum) were frozen with OCT mounting compound (VWR International Ltd., Lutterworth, UK) in isopentane cooled in liquid nitrogen. Pectoral and gastrocnemius muscle transverse cryosections were taken at the mid-belly region of each tissue at 12 μm using a Bright Crysotat at −20 °C (Bright Instruments, Huntingdon, UK). Sections were adhered to Superfrost slides (Fisher Scientific, Loughborough, UK); air dried and stored at −80 °C. Prior to staining, slides were allowed to thaw at room temperature. Muscles from the right side were stored in an RNA stabilisation solution (RNAlater®; Ambion, Cambridgeshire, UK) at −20 °C pending total RNA extraction and quantitative real-time RT-PCR.

### 2.3 Histology and immunostaining

Pectoral and gastrocnemius sections (ED13 to 18) from the mid-belly region were stained using 0.1% Mayer's haematoxylin and 1% eosin for quantification of total nuclei number, muscle fibre number and mean fibre cross-sectional area as previously described (Heywood et al., 2005).

For nuclear expression of myogenin, pectoral and gastrocnemius sections (on ED18) were fixed in 4% paraformaldehyde and blocked in 5% goat serum. The primary rabbit polyclonal Myogenin M-225 antibody (Santa Cruz Biotechnology, Inc., USA) was applied followed by the secondary horseradish peroxidase (HRP)-conjugated goat anti-rabbit polyclonal antibody (Dako, Denmark) and diaminobenzidine (DAB) as a chromogen.

### 2.4 Image analysis

Light photomicrographs were taken using a Leica light microscope and images were analysed using the Kontron KS300 image analysis software (Zeiss, Oberkochen, Germany). For measurement of entire muscle (pectoral and gastrocnemius) cross-sectional area (*n*=4 muscles/strain/age), low magnification pictures from the mid-belly regions (final magnification×50) were taken. For quantification of total nuclei numbers per cross-sectional area, high magnification pictures (final magnification×400) were taken choosing 4 frames at random from each muscle section such that over 40% of the whole section was counted. Nuclei density numbers were then multiplied by the cross-sectional area values to determine the total number of nuclei in the whole cross-section. Muscle fibre area was measured in ∼200 cells per frame (magnification×400) and the average was taken.

For quantification of total myogenin^+^ nuclei numbers in muscle (pectoral and gastrocnemius) sections, high magnification pictures (final magnification×400) were taken choosing 4 frames at random from each muscle section. In each frame the total number of stained nuclei was counted and averages of the 4 frames then taken; numbers were then represented as total myogenin^+^ nuclei across the entire muscle cross-sectional area.

### 2.5 RNA extraction

A standard acid guanidium thiocyanate-phenol-chloroform extraction method (Chomczynski and Sacchi, 1987) was employed for the purification of total RNA. All reagents were purchased from Sigma UK Ltd. (Poole, UK) unless otherwise stated. Briefly, tissues were homogenised in Tri Reagent according to manufacturers' instructions. Upon aqueous phase separation RNA was precipitated in isopropanol and washed in 75% (v/v) ethanol. The RNA pellet was re-suspended in 50 μl of nuclease-free water followed by DNase treatment (RQ1; Promega UK Ltd.). RNA quantity and quality were evaluated using the NanoDrop® ND-1000 spectrophotometer (Nanodrop Technologies, Wilmington, DE, USA). Total RNA integrity was verified by formaldehyde gel electrophoresis ensuring that the 18 and 28S ribosomal RNA bands were intact under UV light.

### 2.6 Reverse transcription and quantitative real-time RT-PCR

All reactions were carried out simultaneously using the same master-mix to prevent variability and amplification inefficiencies between samples. First strand cDNA synthesis was performed on 1 μg total RNA using the QuantiTect Reverse Transcriptase kit (QIAGEN Ltd., Sussex, UK) following manufacturers' instructions.

Primer sequences and Entrez accession numbers for MyoD, PCNA, myogenin and IGF-I are outlined in Table 1. Primers were designed using the Primer-3 Web-Software (Whitehead Institute for Biomedical Research, MA, USA) and manufactured by MWG-Biotech (Ebersberg, Germany).

**Table 1:** Primer sequences (forward and reverse) used in real-time PCR analyses for MyoD, PCNA, myogenin and IGF-I mRNA expression.

**Target**	**Accession number**	**Forward primer (5′ to 3′)**	**Reverse primer (5′ to 3′)**	**Amplicon size (base pairs)**
**MyoD**	NM_204214	act aca cgg att cac caa atg acc	ccc ttc agc tac agc ttc agc	146
**PCNA**	NM_204170	gag acc tca gcc aca ttg gt	agt cag ctg gac tgg ctc at	173
**Myogenin**	NM_204184	cag agg ttt tac gat ggg ca	cag agt gct gcg ttt cag ag	291
**IGF-I**	NM_001004384	ccc aga aac act gtg tgg tg	att ccc ttg tgg tgt aag cg	126

Real-time RT-PCR assays were conducted using the QuantiTect SYBR Green kit (QIAGEN Ltd., Sussex, UK) following manufacturers' instructions. Reactions were carried out in duplicate and consisted of either 1 μl sample cDNA or serially-diluted DNA standard (target sequence of interest). Samples were processed with a MJ Research Chromo4 real-time PCR detector (Bio-Rad Laboratories Ltd., Hampshire, UK) with the following thermal cycling conditions: 15 min Hotstart at 95 °C, followed by 35 amplification cycles of 15 s denaturation at 94 °C, 25 s annealing at 54 °C and 15 s extension at 72 °C. Quantification analysis was carried out using the MJ Research Opticon 3.1 software from standard curves with correlation coefficient (*r*^2^) greater than 0.98. Gene expression data was normalised to total RNA and presented as copy numbers. Specificity and purity of amplificons were verified from melting curves, agarose gel electrophoresis and DNA sequencing using the Applied Biosystems 3730 DNA analyser (Geneservice Ltd., Oxford, UK).

### 2.7 Statistical analysis

Using GraphPad Prism version 5.00 (GraphPad Software, California, USA) data was analysed by one-way analysis of variance (ANOVA) and, a significant *F* ratio (*P*<0.05) was obtained, *post hoc* Bonferroni tests were performed. Pair-wise comparisons of muscle characteristics or mRNA expression levels were carried out (a) between strains on each ED and (b) within each strain across on various EDs. Numerical values are presented graphically as means±S.E.M. of four replicate chicks per strain per ED.

## 3 Results

### 3.1 Physical activity *in ovo*

**Fig. 1:**
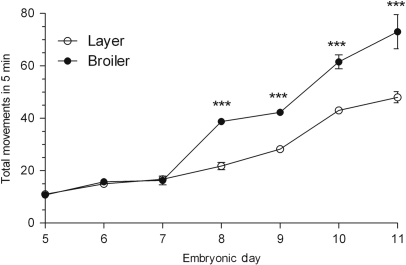
Total number of body movements made *in ovo* over a 5 min period by layer and broiler chicks aged embryonic days (EDs) 5 to 11. Results are presented as means±S.E.M. (*n*=4 animals per strain per day); 3 asterisks represent significant differences at *P*<0.0001 between strains on one particular sample day.

The total number of *in ovo* body movements was measured daily over a 5 min period from ED5 to 11 (Fig. 1). The broilers moved more than the layers from ED8 (1.8-fold, *P*<0.0001), which continued until ED11 (1.5-fold, *P*<0.0001).

### 3.2 Whole body and tissue weights

**Fig. 2:**
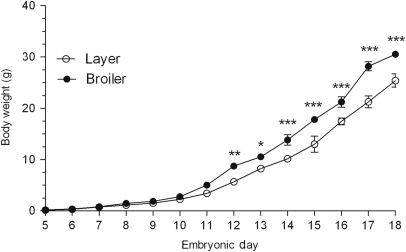
Whole body weight (g) of layer and broiler chicks aged embryonic days (EDs) 5 to 18. Results are presented as means±S.E.M. (*n*=4 animals per strain per day); 1, 2 or 3 asterisks represent significant differences at either *P*<0.05, 0.01 or 0.0001, respectively, between strains on one particular sample day.

**Fig. 3:**
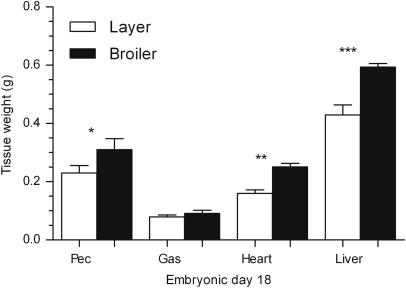
Weights (g) of the pectoral muscle, gastrocnemius muscle, heart and liver of layer and broiler chicks aged embryonic day (ED) 18. Results are presented as means±S.E.M. (*n*=4 animals per strain per day); 1, 2 or 3 asterisks represent significant differences at either *P*<0.05, 0.01 or 0.0001 between strains on one particular sample day. Pec: Pectoral; Gas: Gastrocnemius.

The broilers were heavier than the layers (Fig. 2) from ED12 (by 1.5-fold, *P*<0.01) until ED18 (1.2-fold, *P*<0.0001). On ED18, the broilers presented greater pectoral muscle (1.3-fold, *P*<0.05), heart (1.6-fold, *P*<0.01) and liver (1.4-fold, *P*<0.0001) weights but gastrocnemius muscle weights did not vary (Fig. 3). Relative to body mass, the broilers exhibited greater ratio of pectoral muscle (1.3-fold, *P*<0.01), heart (1.3-fold, *P*<0.05) and liver (1.2-fold, *P*<0.0001) compared to the layers. Gastrocnemius muscle weight relative to body mass did not vary between strains.

### 3.3 Levels of MyoD, PCNA, myogenin and IGF-I mRNAs in the pectoral muscle

**Fig. 4:**
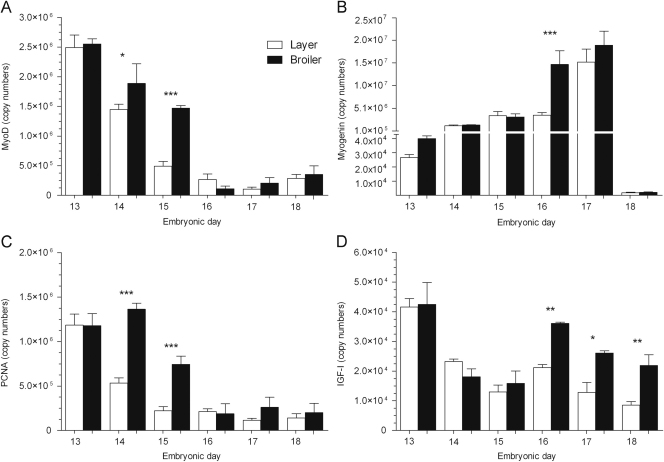
Real-time PCR analyses on 1 μg of total RNA extracted from the pectoral muscles of layer and broiler chicks aged embryonic days (EDs) 13–18. The mRNA expressions (copy numbers) for (A) MyoD, (B) PCNA, (C) myogenin and (D) IGF-I are shown. Results are presented as means±S.E.M. (*n*=4 animals per strain per day); 1, 2 or 3 asterisks represent significant differences at either *P*<0.05, 0.01 or 0.0001, respectively, between strains on one particular sample day.

The mRNA levels of MyoD (Fig. 4A) decreased from ED13 to 14 (1.7-fold, *P*<0.0001) and ED14 to 15 (3-fold, *P*<0.0001) in the layers. Between ED15 and 18 MyoD levels did not vary in the layers. In the broilers, MyoD levels decreased from ED13 to 14 (1.3-fold, *P*<0.05) and from ED15 to 16 (13.5-fold, *P*<0.0001). Between ED16 and 18 MyoD levels were comparably low in the broilers. The mRNA levels of MyoD were higher in the broilers on ED14 (1.3-fold, *P*<0.05) and 15 (3-fold, *P*<0.0001) compared to the layers.

The expression profile of PCNA (Fig. 4B) was similar to that of MyoD, in that PCNA mRNA levels were reduced from ED13 to 14 (2.2-fold, *P*<0.0001) and ED14 to 15 (2.4-fold, *P*<0.05) before remaining equally low between ED15 to 18 in the layers. In the broilers, PCNA levels decreased from ED14 to 15 (1.8-fold, *P*<0.0001) and ED15 to 16 (4-fold, *P*<0.0001); levels subsequently remained low between ED16 and 18. The mRNA levels of PCNA were higher in the broilers on ED14 (2.5-fold, *P*<0.0001) and 15 (3.4-fold, *P*<0.0001) compared to the layers.

The mRNA levels of myogenin (Fig. 4C) increased from ED13 to 17 in layers (576-fold, *P*<0.0001) and broilers (474-fold, *P*<0.0001) and thereafter abruptly declined to equally low levels in all of the ED18 chicks. Myogenin levels were higher in the broilers on ED16 (by 4.1-fold, *P*<0.0001) compared to the layers.

The mRNA levels of IGF-I (Fig. 4D) initially decreased from ED13 to 15 in both layers (3.2-fold, *P*<0.0001) and broilers (2.7-fold, *P*<0.0001). IGF-I levels subsequently increased in broilers (2.3-fold, *P*<0.0001) from ED15 to 16 (only trend in layers) before declining once again until ED18. IGF-I mRNA levels were greater in the broilers on ED16 (by 1.7-fold, *P*<0.01), 17 (by 2-fold, *P*<0.05) and 18 (by 2.6-fold, *P*<0.01) compared to the layers.

### 3.4 Levels of MyoD, PCNA, myogenin and IGF-I mRNAs in the gastrocnemius muscle

**Fig. 5:**
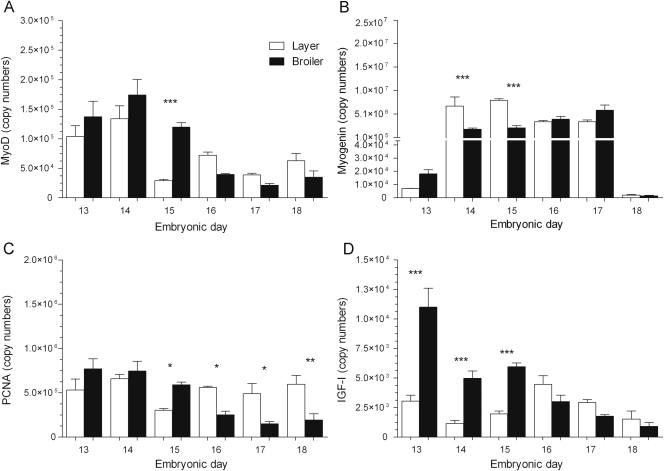
Real-time PCR analyses on 1 μg of total RNA extracted from the gastrocnemius muscles of layer and broiler chicks aged embryonic days (EDs) 13–18. The mRNA expressions (copy numbers) for (A) MyoD, (B) PCNA, (C) myogenin and (D) IGF-I are shown. Results are presented as means±S.E.M. (*n*=4 animals per strain per day); 1, 2 or 3 asterisks represent significant differences at either *P*<0.05, 0.01 or 0.0001, respectively, between strains on one particular sample day.

The mRNA levels of MyoD (Fig. 5A) decreased from ED14 to 15 in the layers (4.6-fold, *P*<0.0001) and from ED14 to 15 (1.5-fold, *P*<0.05) then ED15 to 16 in the broilers (3-fold, *P*<0.01). MyoD levels remained similarly low in each of the strains from then on until ED18. MyoD levels were greater in the broilers on ED15 (by 3-fold, *P*<0.0001) compared to the layers.

The mRNA levels of PCNA (Fig. 5B) decreased from ED14 to 15 in the layers (2-fold, *P*<0.01) and from ED15 to 16 in the broilers (2.3-fold, *P*<0.001). PCNA levels were subsequently raised in the layers (1.9-fold, *P*<0.05) but reduced in the broilers (2.3-fold, *P*<0.01) from ED15 to 16. PCNA levels were greater in the broilers on ED15 (by 1.9-fold, *P*<0.05) compared to the layers yet greater in the layers on ED16 (2.2-fold, *P*<0.05), 17 (3.2-fold, *P*<0.05) and 18 (3.1-fold, *P*<0.01) compared to the broilers.

The mRNA levels of myogenin (Fig. 5C) greatly increased from ED13 to 14 by over 1000-fold, (*P*<0.0001) in layers and by over 300-fold, (*P*<0.0001) in broilers. Myogenin levels were greater in the layers on ED14 (by 3.6-fold, *P*<0.0001) and 15 (by 5-fold, *P*<0.0001) compared to the broilers.

The mRNA levels of IGF-I (Fig. 5D) increased from ED15 to 16 (2.3-fold, *P*<0.01) and then decreased from ED16 to 18 (2.9-fold, *P*<0.0001) in the layers. In the broilers, IGF-I levels decreased between ED13 and 18 (12-fold, *P*<0.0001). IGF-I levels were greater in the broilers on ED13 (3.6-fold, *P*<0.0001), 14 (4.3-fold, *P*<0.0001) and 15 (3-fold, *P*<0.0001) compared to the layers.

### 3.5 Pectoral and gastrocnemius muscle cellular parameters

**Fig. 6:**
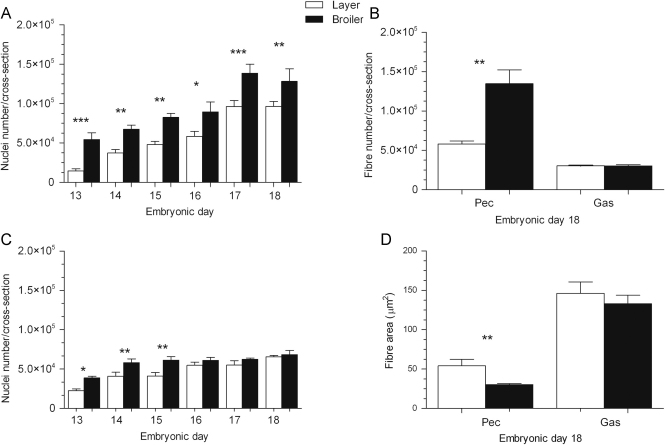
Total number of nuclei measured across the entire cross-sectional area of the (A) pectoral and (B) gastrocnemius muscles of layer and broiler chicks aged embryonic days (EDs) 13–18. The total (C) number of muscle fibres and (D) mean fibre area (μm^2^) measured across the entire cross-sectional area of pectoral and gastrocnemius muscles of layer and broiler chicks aged ED18. Results are presented as means±S.E.M. (*n*=4 animals per strain per day); 1, 2 or 3 asterisks represent significant differences at either *P*<0.05, 0.01 or 0.0001, respectively, between strains on one particular sample day. Pec: Pectoral; Gas: Gastrocnemius.

The entire cross-sectional area of the pectoral muscle was higher in broilers on ED13 compared to layers (2.7-fold, *P*<0.05) yet there were no differences between strains on ED18 (Data not shown). The gastrocnemius muscle cross-sectional areas did not vary between strains on ED13 or 18. The total apparent number of nuclei measured in the entire pectoral muscle cross-section (Fig. 6A) increased from ED13 to 17 in both layers (6.6-fold, *P*<0.0001) and broilers (2.4-fold, *P*<0.0001). Nuclei numbers did not vary from ED17 to 18 in each of the strains. On each of the days (between ED13 and 18) nuclei numbers were greater in the broilers (by 3.7-fold on ED13, *P*<0.0001 and 1.3-fold on ED18, *P*<0.01) compared to the layers. The total apparent number of nuclei measured in the entire gastrocnemius muscle cross-section (Fig. 6B) increased from ED13 to 16 in layers (2.4-fold, *P*<0.0001) and from ED13 to 14 in broilers (1.5-fold, *P*<0.01). From then onwards, (i.e. from ED16 to 18 in layers and ED14 to 18 in broilers) nuclei numbers remained similar between layers and broilers among stages of age. Nuclei numbers were greater in the broiler on ED13 (1.7-fold, *P*<0.05), 14 (P1.4-fold, <0.01) and 15 (1.5-fold, *P*<0.01) compared to the layers.

The total apparent number of fibres (Fig. 6C) measured in the entire pectoral muscle cross-section on ED18 was greater (2-fold, *P*<0.01) in broilers than in layers. Furthermore, these broiler fibres (Fig. 6D) had smaller fibre cross-sectional areas (2-fold, *P*<0.01). The total apparent number of gastrocnemius muscle fibres (Fig. 6C) and mean fibre area (Fig. 6D) did not differ between broilers and layers on ED18.

### 3.6 Myogenin immunostaining in the pectoral and gastrocnemius muscles

**Fig. 7:**
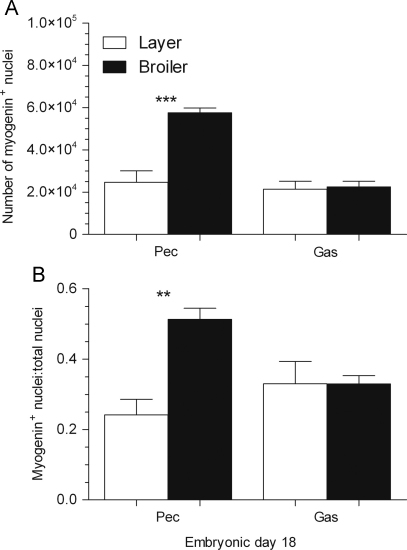
Total number of myogenin^+^ nuclei measured across the entire cross-sectional area (A) and ratio of myogenin^+^ nuclei over total nuclei numbers (B) in the pectoral (Pec) and gastrocnemius (Gas) muscles of layer and broiler chicks aged embryonic day (ED) 18. Results are presented as means±S.E.M. (*n*=4 animals per strain per day); 3 asterisks represent significant differences at *P*<0.0001 between strains on ED18.

The total apparent number of myogenin^+^ nuclei (Fig. 7A) measured in the entire muscle cross-section on ED18 was greater (2.3-fold, *P*<0.0001) in the pectoral muscle of broilers than in that of layers but there was no difference in the gastrocnemius muscle. In the pectoral muscle, the ratio of myogenin^+^ nuclei over total nuclei number was greater in broilers than layers (2.1-fold, *P*<0.01) whereas this ratio was not significantly different between strains in the gastrocnemius muscle on ED18 (Fig. 7B).

## 4 Discussion

In the poultry industry, chickens have been genetically selected for different traits. In the meat-type broilers, as opposed to egg-layers, selection pressure has been placed on rapid post-hatch growth rates and enhanced pectoral muscle mass. Other muscles in their body, including the leg gastrocnemius, which is important for postural stability, have not been directly selected for (Arthur and Albers, 2003). Rapid growth rates have been correlated with greater incidence of leg disorders and impaired ability to walk (Kestin et al., 2001, Vestergaard and Sanotra, 1999) thereby causing welfare issues, which warrant interventions. In an attempt to explain how genetic selection has promoted breast but not leg muscle differentiation and growth, we hypothesise that the rapid post-hatch growth rate of broilers is associated with an earlier state of embryonic hyperactivity with differing outcomes on breast and leg muscle development.

### 4.1 Increased broiler activity *in ovo* linked to increased pectoral but not gastrocnemius muscle weight

Embryonic activity has been highlighted as a contributing factor to musculoskeletal development. For instance, studies using layer embryos, which had been subjected to a transient increase in incubation temperature during the early stages of embryogenesis demonstrated an increase in total body movements *in ovo* and greater leg myofibre numbers and muscle mass by ED18 (Hammond et al., 2007). Embryonic hyperactivity more specifically induced by treatment with the neuromuscular stimulant 4-aminopyridine (4-AP) has also been shown to promote body weight gain as well as muscle cell proliferation and fibre hyperplasia (McEntee et al., 2006, Heywood et al., 2005). Conversely, paralysis of early to mid-stage layer embryos impedes subsequent skeletal muscle growth (Hall and Herring, 1990). In the present study, the broiler chicks produced a greater number of total body movements *in ovo* compared to the layers; these changes were noted from ED8 until 11. Due to spatial constraints inflicted by the limited shell size on the growing embryo, these movements were not quantified after ED11. Increased broiler movement was accompanied by increased whole body weight from ED12 to ED18. On ED18, the broilers additionally presented greater pectoral muscle, heart and liver weights but the weight of the gastrocnemius muscle was comparable to that of layer chickens implying that broilers breast and limb muscle mass was differently affected by increased embryonic activity. The mechanism by which increased activity promotes skeletal muscle growth appears to involve the IGF system. Embryonic 4-AP treatment *in ovo* as well as electrical stimulation *in vitro* affect the myogenic expression levels of IGFs and their binding proteins (McEntee et al., 2006, Bayol et al., 2005). Furthermore, the IGF system is a well characterised mediator of mechanical signal transduction leading to fibre hypertrophy and growth in adult skeletal muscle (Tidball, 2005). In light of this, we sought to establish whether the strain-dependant changes in embryonic activity observed would result in alterations in the expression patterns of myogenic factors and IGF-I and given that increased embryonic movement in broilers had differing outcomes on breast and leg muscle mass we sought to determine whether the ontogenic expression of such factors was affected in a muscle specific manner.

### 4.2 Evidence of increased myoblast mitotic activity in the pectoral and gastrocnemius muscle of broilers up to ED15

The expression of MyoD, a primary MRF expressed in proliferating myoblasts (Weintraub, 1993), was higher in broilers on ED14–15 in the pectoral and on ED15 in the gastrocnemius muscles before reaching layer-levels from ED16 onwards, in both muscles. As expected, increased MyoD mRNA expression was accompanied by increased expression of PCNA mRNA, whose rate of synthesis is directly correlated with the proliferative rate of cells (Bravo et al., 1987, Kohler et al., 2005), in both muscles of broilers namely on ED14/15 in the pectoral and on ED15 in the gastrocnemius muscle. Increased MyoD and PCNA transcriptions on ED15 are indicative of higher myogenic cell commitment and proliferation in both the pectoral and gastrocnemius muscles of broilers compared with layers. This was confirmed by histological analysis showing increased nuclei numbers in broilers versus layers in both muscles up to ED15. However, we note that broiler MyoD and PCNA mRNAs were expressed at higher levels over a longer period (ED14–15) in the pectoralis than in the gastrocnemius muscle (ED15) compared with layers implying a relative reduction in cell proliferation and myogenic commitment in the gastrocnemius versus pectoral muscle in this strain which may help explain subsequent observations. Despite evidence of increased cell proliferation (raised nuclei counts and PCNA) and commitment to the myogenic lineage (raised MyoD) in both pectoral and gastrocnemius muscle of broilers at ED15, muscle specific differences followed. From ED16 to ED18, nuclei counts continued to rise in the pectoral muscle of broilers versus layers, whereas nuclei counts in the gastrocnemius were similar to that of layers from ED16 to 18 with concomitantly unchanged MyoD mRNA levels in the gastrocnemius muscle of broilers.

### 4.3 Evidence of increased myogenic differentiation in the pectoral but not in the gastrocnemius muscles of broilers

The increased number of nuclei in the pectoral muscle of broilers at ED18 was accompanied by increased number of muscle fibres of smaller diameters indicative of increased packing density, which may increase the potential for post-hatch hypertrophy and weight gain. In contrast, total gastrocnemius fibre numbers were similar between broilers and layers on ED18, which coincides with similar nuclei numbers between strains in this muscle. In order to understand why the rate of nuclear accretion and fibre formation did not increase in the broiler gastrocnemius muscle from ED15 like it did in the pectoral muscle, we examined the temporal mRNA expression patterns of myogenin (defining the progression of differentiation) and IGF-I (important in both proliferation and differentiation).

Myogenin is a direct transcriptional target of MyoD; its activation involves the binding of MyoD to noncanonical E boxes through interaction with resident heterodimers of the HOX-TALE in the promoter region of myogenin (Berkes et al., 2004). In the pectoral muscle, myogenin mRNA expression levels peaked earlier (ED16) in broilers than in layers (ED17). As myogenin expression is known to precede cell cycle withdrawal (Andres and Walsh, 1996), it is possible that exit from the cell cycle began at an earlier stage in the broiler pectoral muscles allowing a longer period of muscle fibre formation. Furthermore, the elevated myogenin levels in the broiler muscles on ED16 (24 hours after elevated MyoD levels) and increased immunostaining for myogenin^+^ nuclei as well as increased proportion of myogenin^+^ on ED18 suggest a greater proportion of myoblasts was signalled to differentiate at this earlier embryonic stage, which would favour increased fibre formation and muscle size. In support of this, the broiler pectoral muscle was heavier than in layers by ED18.

Expression of myogenin in the gastrocnemius muscles reflected a different scenario. Broiler myogenin levels did not surpass layer-levels on any of the developmental stages examined, regardless of elevated MyoD levels on ED15. Immunostaining for myogenin^+^ nuclei also revealed that there were no differences in the number and proportion of positively stained nuclei in the broiler and layer gastrocnemius muscles on ED18. Consequently, muscle weights did not differ between broilers and layers at this time. Although myogenin mRNA levels were notably higher in the layer gastrocnemius muscles on ED14 and 15 this did not culminate in increased muscle hyperplasia by ED18.

### 4.4 Muscle specific differences in IGF-I expression patterns in broilers linked to differing outcomes for myogenic differentiation

IGF-I plays a key role in the mechanical signal transduction of skeletal muscle growth (Tidball, 2005). IGF-I is also critical for normal skeletal muscle development, with crucial roles in proliferation, differentiation and growth of muscle cells (Duclos, 2005). More specifically, IGF-I exerts opposing time dependant effects on myoblasts whereby it initially inhibits differentiation to promote proliferation and subsequently promotes cell cycle withdrawal and differentiation. These opposing IGF-I actions are mediated by initial inhibition and subsequent stimulation of myogenin transcription, which are regulated at the promoter level through the Erk1/2MAPK signalling pathway (Adi et al., 2000, Adi et al., 2002). In the present study, consistent with the increased embryonic activity measured in broilers, muscle IGF-1 mRNA was also raised in both pectoral and gastrocnemius muscle of broilers. However, we note muscle specific differences in the ontogenic timing of IGF-I up-regulation between strains. IGF-I mRNA levels were higher in broiler pectoral muscle compared with layers during the later stages of myogenesis examined, namely ED16 and 18, and this was concomitant with myogenin upregulation on ED16 suggesting that myogenic differentiation and growth may have been maximally stimulated by IGF-I at this stage. This is supported by greater pectoral muscle mass at ED18.

In the gastrocnemius muscle of broilers, IGF-I mRNA levels were increased during the earlier stages of myogenesis examined namely from ED13 to 15 and subsequently, myogenin levels in broilers did not exceed those of layers on any of the days tested (regardless of raised MyoD on ED15). Given the role of IGF-I in regulating the timing of myogenin transcription (Adi et al., 2002), we propose that the premature upregulation of IGF-I in the gastrocnemius muscle of broilers, at a time when myoblasts were rapidly proliferating (ED13–15) had a more potent inhibitory effect on myogenin transcription and may have prevented its subsequent upregulation as was observed in the pectoral muscle on ED16 as well as the associated increase in myogenic differentiation and growth.

Results from the present study have shown that genetic selection for enhanced growth in chickens has affected embryonic activity and the temporal expression patterns of IGF-I and MRFs involved in cell proliferation and differentiation, in a muscle specific manner. In both the selected pectoral and unselected gastrocnemius muscles of broilers, we note a rise in cell proliferation (nuclei numbers) between ED13 and 15 yet muscle specific differences follow. In the broiler pectoral muscles, the rise in cell proliferation on ED13 and 15 is followed by increased MyoD levels on ED14/15, closely followed by upregulated myogenin levels on ED16 and IGF-I on ED16, 17 and 18. These changes are indicative of greater myogenic differentiation and growth which help explain the exacerbated growth of the pectoral muscle in the broilers. However, in the gastrocnemius muscles of broilers, the higher levels of MyoD on ED15 were not followed by any subsequent changes to myogenin and IGF-I levels. In fact, IGF-I was upregulated between ED13 and 15, at a time when proliferation was high, which is presumed to have an inhibitory effect on myogenin transcription and hence helps explain why the exacerbated differentiation and growth observed in the pectoral muscle of broilers did not occur in the gastrocnemius muscle.

### 4.5 Implications for the broiler industry

Broilers are at the forefront of mounting welfare problems, typically suffering from sudden death syndrome, leg disfigurements and impaired locomotion (for review, see Julian, 2005). Understanding the diverging myogenic developmental mechanisms in leg and breast muscles resulting from intense genetic selection may enable the development of welfare intervention strategies aiming at promoting leg muscle growth in broilers. Manipulating the developmental onset of key factors regulating muscle growth, possibly through egg incubation regimes may allow for increased leg muscle mass and hence allow broiler chickens to better support their rapidly increasing body weight and reduce the incidence of post-hatch abnormalities. This is currently being investigated.
